# Introns in *Cryptococcus*

**DOI:** 10.1590/0074-02760170519

**Published:** 2018-02-19

**Authors:** Guilhem Janbon

**Affiliations:** Institut Pasteur, Unité Biologie des ARN des Pathogènes Fongiques, Département de Mycologie, Paris, France

**Keywords:** intron, Cryptococcus, RNA, yeast, alternative splicing

## Abstract

In *Cryptococcus neoformans*, nearly all genes are interrupted by small introns. In recent years, genome annotation and genetic analysis have illuminated the major roles these introns play in the biology of this pathogenic yeast. Introns are necessary for gene expression and alternative splicing can regulate gene expression in response to environmental cues. In addition, recent studies have revealed that *C. neoformans* introns help to prevent transposon dissemination and protect genome integrity. These characteristics of cryptococcal introns are probably not unique to *Cryptococcus*, and this yeast likely can be considered as a model for intron-related studies in fungi.

Identified for the first time at the end of the 19th century by Sanfelice in fruit juice (Sanfelice 1894), *Cryptococcus* is now recognised as a major fungal human pathogen responsible for thousands of deaths every year around the world (Rajasingham et al. 2017). There are two main lineages of pathogenic *Cryptococcus*, referred to collectively as *Cryptococcus* complex. Whereas *Cryptococcus neoformans* affects immunocompromised patients and exists as two varieties (i.e., var*. neoformans* and var. *grubii*), *Cryptococcus gattii* exists in several sub-lineages, some of which can infect immunocompetent patients (Kwon-Chung et al. 2015). Because *Cryptococcus* lives in the environment (mainly in soil and decaying wood), its biology has been shaped through its interactions with environmental microbiota, as well as seasonal modifications in the physical and chemical soil parameters (May et al. 2016). Much probably, the main *Cryptococcus* virulence factors, which include capsule and melanin production, have been selected to resist natural environmental predators, such as amoebae and worms (May et al. 2016).

*Cryptococcus* cells mostly grow as *Saccharomyces cerevisiae*-like haploid budding yeast, although filamentous or very large multiploid cells can be observed *in vitro* and *in vivo* (Zaragoza & Nielsen 2013). Despite this morphological similarity, *Cryptococcus* biology and genetics are, in many aspects, different from *S. cerevisiae*, due in part to the specificity of virulence-associated attributes of *C. neoformans*. For instance, the metabolic pathways and genes required to synthesize polysaccharide capsule and melanin are not present in *S. cerevisiae* (Moyrand et al. 2007, Alspaugh 2015). *Cryptococcus* biological specificity also resides in its complex and dynamic transcriptome structure, which in this opportunistic pathogen can be viewed as a method to adapt to different environments. Introns are probably the most prominent characteristic of the cryptococcal genes. Whereas a typical *S. cerevisiae* gene is intronless, nearly all *Cryptococcus* genes contain multiple introns (Figure). Recently, these introns and their splicing have been shown to play major roles in gene expression and the control of genetic instability in this pathogenic yeast. Here, I review the current literature surrounding intron structural characteristics and evolutionary conservation, as well as the roles of introns in the *Cryptococcus* biology.

**Figure:**
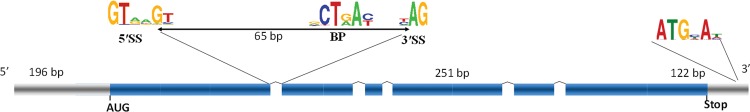
Schematic representation of a typical *Cryptococcus* gene. The coding sequence (CDS) is represented in blue whereas the untranscribed regions (UTR) are in grey. The consensus sequences associated with the 5’ splice site (5’SS) 3’ splice site (3’SS) and branching point (BP) are indicated. The average size of the introns, exons, 3’ and 5’UTR regions are given.

*Intron position and structure* - Historically, the early cloning and sequencing of *Cryptococcus* genes revealed the presence of multiple introns. In 1990, Edman and Kwon-Chung cloned the gene encoding *C. neoformans* orotidine monophosphate pyrophosphorylase (*URA5*) and identified two small introns of 52 and 49 nucleotides (Edman & Kwon-Chung 1990). The gene encoding dihydrofolate reductase was cloned three years later by JC Edman and displayed a similar structure with two short introns (Sirawaraporn et al. 1993). All *Cryptococcus* genes subsequently cloned contain at least one intron, suggesting that all genes in this pathogenic yeast contain at least one intron. Of note, the phosphoribosylanthranilate isomerase-encoding gene (*TRP1*) was originally reported to be intronless (Perfect et al. 1992) but does indeed contain two short introns within its sequence (http://fungidb.org/fungidb/app/record/gene/CNAG_04501) (Stajich et al. 2012). Large-scale sequencing of cDNA molecules and the more recent use of RNA-Seq data confirmed the complex structure of cryptococcal genes (Kupfer et al. 2004, Loftus et al. 2005, Janbon et al. 2014, González-Hilarion et al. 2016, Ferrareze et al. 2017). Each gene contains an average of 5.7 introns. These introns are mostly present in coding sequence (CDS), although some are present in the 5’ and 3’ UTR regions. This is in sharp contrast to *S. cerevisiae*, in which only 283 genes contain an intron (Juneau et al. 2007, Janbon et al. 2014). In *C. neoformans* var. *grubii*, introns also have been identified in transcribed active regions with no obvious coding potential (miscRNAs) (Janbon et al. 2014). Overall, the *C. neoformans* genomes contain more than 40,000 introns. With 3.35 introns per kb of coding sequence, *C. neoformans* is the most intron-rich fungal species for which a reliable genome annotation is available (Stajich et al. 2007, Janbon et al. 2014). It is important to note here that the presence of introns in fungal genes is probably more the rule than the exception. In that sense, *C. neoformans* can be considered to be a model for intron-related studies in fungi (Stajich et al. 2007).

As in all fungi, *Cryptococcus* introns are relatively small, with an average size of 65 nt; the UTR introns are slightly larger than those in the CDS. The analysis of *C. neoformans* and *C. gattii* intron length suggests strong but complex evolutionary selection of intron size (Hughes et al. 2008, Janbon et al. 2014); thus, the size of UTR introns, as well as the first and last introns within the CDS, seem to be constrained by different selective pressures, suggesting potential specific roles or splicing patterns (Hughes et al. 2008). As shown in Figure, *Cryptococcus* introns are associated with specific consensus motifs with very minor differences between species (Kupfer et al. 2004, Irimia et al. 2009, Janbon et al. 2014). However, in contrast to *S. cerevisiae*, in which splicing signals conform closely to consensus, signals in *C. neoformans* are more degenerated*,* resembling metazoan introns in this respect. Interestingly, a metazoan-like bias in amino acid and codon usage at the *C. neoformans* 3’ and 5’ exon ends has been also observed (Warnecke et al. 2008). Again, this bias was not observed in *S. cerevisiae* or *Schizosaccharomyces pombe*, suggesting specific exon-based splicing regulation in *C. neoformans*. Consistent with this model, *C. neoformans* proteome contains multiple homologues of SR or SR-like proteins known to be involved in splicing an alternative splicing regulation in metazoans but not typically found in single cell eukaryotes [(Warnecke et al. 2008, Dumesic et al. 2013); Janbon, unpublished observations]. Although mechanistic analyses are yet to be performed, it is tempting to put these structural observations in perspective with the abundance and complex regulation of alternative splicing recently reported in *C. neoformans* (González-Hilarion et al. 2016).

*Intron gain and loss* - The evolutionary dynamics of introns have been widely studied in fungi, including *C. neoformans* (Nielsen et al. 2004, Sharpton et al. 2008, Croll & McDonald 2012). These studies, all based on genome sequence analyses, identified few examples of intron gain and loss between the *Cryptococcus* lineages (Roy 2009, Croll & McDonald 2012). Stajich and Dietrich identified a *C. neoformans* var. *grubii* gene from which 10 adjacent introns have been lost, suggesting an mRNA-based mechanism (Stajich & Dietrich 2006). Conversely, in 2009 Row found evidence for five cases of intron gain in the *C. neoformans* var. *neoformans* genome (Roy 2009). These are recent events, as *C. neoformans* var. *grubii* and *C. neoformans* var. *neoformans* diverged only 37 million years ago (Kwon-Chung et al. 2015). The recent genome re-annotation of the two *C. neoformans* varieties based on RNA-Seq data gave us the opportunity to gain insight into intron dynamics. We identified 6,241 orthologous gene couples and analysed intron conservation between the varieties (González-Hilarion et al. 2016). Whereas half of the introns in the 5’UTR and 90% in the 3’UTR were either lost or at a different position, the overwhelming majority (99.5%) of introns in the CDS were present in the same position in both *C. neoformans* varieties, confirming results of previous studies (Roy 2009, Croll & McDonald 2012, González-Hilarion et al. 2016). Overall, these data revealed that intron gain and loss are very rare events, suggesting a strong selective pressure to maintain intron richness in *Cryptococcus* genes.

*Biological importance* - Introns play a central role in the regulation of gene expression in *C. neoformans*, which may explain the strong selective pressure in favor of introns in this species. Indeed, *in situ* replacement of a wild-type multi-exonic gene by an intronless version of the same gene is usually associated with a strong decrease in gene expression (Dumesic et al. 2013, Goebels et al. 2013). In some cases, intron deletion completely abolishes gene expression (Goebels et al. 2013). Our analysis on a model gene suggests that most introns play a cumulative positive role probably because their splicing promotes mRNA export out of the nucleus (Goebels et al. 2013); in the absence of introns, mRNA molecules would be poorly exported and instead degraded by nuclear exonucleases. It is interesting to note that only 35 expressed genes in *C. neoformans* var. *grubii* are intronless, further supporting the idea that introns play a central role in the regulation of gene expression (Janbon et al. 2014). Furthermore, indirect evidence suggests that incompletely spliced mRNA molecules remain in the nucleus, where they are degraded (González-Hilarion et al. 2016). Accordingly, poorly spliced introns play a negative role in the regulation of gene expression (Goebels et al. 2013), and incompletely spliced mRNAs containing a premature termination codon are not degraded by the nonsense-mediated mRNA decay cytoplasmic pathway (González-Hilarion et al. 2016). Interestingly, genome-wide and single-gene studies revealed that alternative splicing (mostly intron retention) affects most *Cryptococcus* genes (Loftus et al. 2005, Missall et al. 2005, González-Hilarion et al. 2016). In addition, RNA-Seq analyses strongly suggest that alternative splicing is tightly and specifically regulated by environmental cues (González-Hilarion et al. 2016). Thus, in *C. neoformans* and probably in most fungi, alternative splicing is certainly not a major source of proteome diversification, but it should be considered an additional layer of gene expression regulation providing a mechanism by which each cell finely tunes gene expression levels in response to environmental modifications (González-Hilarion et al. 2016).

Introns and intron splicing prevent genomic instability in *C. neoformans.* For example, intron splicing efficiency regulates the expression of transposon-related genes in *C. neoformans*. Dumesic and colleagues demonstrated that inefficiently spliced mRNAs, which are typically transcribed based on transposable sequences, are targeted by the Spliceosome-Coupled and Nuclear RNAi complex (SCANR), becoming a template for dsRNA synthesis and siRNA production (Dumesic et al. 2013). Accordingly, this RNAi-dependent nuclear mechanism also involves the lariat debranching enzyme Dbr1. Dumesic and colleagues also reported that transposon-related transcripts are more likely to be associated with the spliceosome, suggesting that their introns are poorly spliced. Thus, *C. neoformans* introns and their splicing limit the expression of transposon-related genes and prevent transposon dissemination in the genome.

Another emerging mechanism by which introns may protect genomes is by preventing DNA-RNA hybrid (R-loop) formation in the wake of the RNA polymerase II, thus avoiding the associated accumulation of DNA damage (Bonnet et al. 2017). Bonnet and colleagues analysed phenotypes associated with deletion of the conserved THO complex component encoding the gene *HPR1* which is known to prevent R-loop formation in *S. cerevisiae* and human cells (Domínguez-Sánchez et al. 2011). They found that this mutation has very little impact on growth and R-loop formation in intron-rich organisms such as *C. neoformans,* but it is associated with a strong growth defect and marked increase of R-loop formation in intron-poor yeasts such as *Candida glabrata* and *S. cerevisiae*. They also showed that introns and spliceosome-dependent mRNP assembly, but not splicing *per se*, prevent R-loop formation and genome instability (Bonnet et al. 2017). Altogether, these studies reveal the central role of introns in *Cryptococcus* species for control of genome stability.

*Summary and future directions* - In summary, these studies have revealed introns as key features in the biology of *Cryptococcus* and, in the maintenance of their genome integrity. They also suggest that their structure have been finely selected throughout evolution. Nevertheless, a number of questions remain to be answered. (1) For instance, the mechanisms regulating intron gain and loss in fungi is still a very active domain of research. A better description of intron structure and position in the different lineages of *C. gattii* and *C. neoformans* (Kwon-Chung et al. 2015) will likely provide insights into this matter. Similarly, although many genomes have been sequenced in recent years (Desjardins et al. 2017), a precise comparison of intron position within each lineage would identify recent intron gain or loss events. In addition, recent reports identified independent links between the biology of introns and genomic stability in *Cryptococcus*, thus paving new avenues for genome structure evolution in fungi. (2) Additionally, the role of introns in gene regulation requires further analyses. The current model on *Cryptococcus* intron retention-dependent regulation of gene expression (González-Hilarion et al. 2016) suggests the existence of complex networks of transduction pathways and RNA binding proteins that precisely regulate alternative splicing in response to environmental cues. However, no elements within these networks have been identified, and deciphering the mechanisms regulating alternative splicing will represent a major challenge in future studies. (3) Furthermore, intron biology is intertwined with *C. neoformans* RNA biology. For example, as previously reported in metazoans several alternative polyadenylation sites within introns have been identified (Janbon et al. 2014). In metazoans, polyadenylation and splicing are there in competition and their alternative usage results in transcripts of different size, stability and sometime coding potential (Tian & Manley 2017). In fungi and more specifically in *C. neoformans* the biological consequences of this choice remain to be studied. The roles of introns in the UTRs of number of *Cryptococcus* genes are mainly unknown. In metazoans, introns in UTRs were ignored for a long time but they have recently been shown to play important and unique roles in the regulation of gene expression (Bicknell et al. 2012). In *Cryptococcus*, the specificity of their structure and evolutionary conservation suggest also unique functions which remains to be identified. (4) Finally, introns have been identified in miscRNAs, but the function, splicing, and evolutionary conservation of this intron subclass have not been studied yet.
